# Genomic analyses of a widespread blueberry virus in the United States

**DOI:** 10.1016/j.virusres.2023.199143

**Published:** 2023-06-07

**Authors:** Daisy Stainton, Dan E.V. Villamor, Andrea Sierra Mejia, Ashish Srivastava, Dimitre Mollov, Robert R. Martin, Ioannis E. Tzanetakis

**Affiliations:** a Department of Entomology and Plant Pathology, Division of Agriculture, University of Arkansas System, Fayetteville, AR 72701, USA; b USDA-ARS, Horticultural Crops Disease and Pest Management Research Unit, 3420 NW Orchard Ave, Corvallis, OR 97330; c Oregon State University, Corvallis, OR 97330, USA

**Keywords:** Vaccinium, Distribution, Genomics

## Abstract

Screening of blueberry accessions using high throughput sequencing revealed the presence of a new virus. Genomic structure and sequence are similar to that of nectarine stem pitting associated virus (NSPaV), a member of the genus *Luteovirus,* family *Tombusviridae*. The full genome of the new luteovirus, tentatively named blueberry virus L (BlVL), was characterized and analyzed. Similar to NSPaV, BlVL does not contain readily identifiable movement proteins in any of the seven isolates sequenced. More than 600 samples collected from five states were screened and 79% were found infected, making BlVL the most widespread blueberry virus in the United States.

## Highlights

• Genomic and evolutionary analysis of blueberry virus (BlVL), a new luteovirus.
• A cryptic ORF is under strong positive selection.
• BlVL is present in almost 80% of the more than 600 samples tested.
• Most widespread blueberry virus in the United States.
• Given the BlVL diversity movement by propagation material is highly unlikely.

## 1 Introduction

Blueberry (*Vaccinium* spp.) is a perennial crop with plants grown in the field for several decades (Retamales and Hancock, 2018). For this reason, there is a high probability that plants become infected by and accumulate viruses; acting as reservoirs for spread within and across fields. There are currently 18 viruses known to infect the crop (Saad et al., 2021; Villamor et al., 2023) with many causing significant losses wherever they occur (Martin and Tzanetakis, 2018).

The genus *Luteovirus* (family *Tombusviridae*) consists of 13 ICTV accepted species (ICTV; https://ictv.global/taxonomy, accessed May 22nd, 2023), infecting both monocots and dicots yet none is known to infect blueberry. Luteoviruses have a monopartite positive-sense RNA genome and are aphid transmissible in a circulative, non-propagative manner (Gray and Gildow, 2003; Ng and Perry, 2004). The majority encode six or seven proteins that are expressed through cap independent translation, frameshifts, readthroughs and subgenomic RNAs. P1 and the P1-P2 replicase are involved in replication whereas P3, the capsid protein (CP) and the P3-P5 fusion (CP with read through domain; CP-RTD) are involved in encapsidation and aphid transmission (Chay et al., 1996). There are two movement proteins (MPs): P3a encodes a small amino acid (aa) protein involved in long-distance movement (Smirnova et al., 2015) whereas P4 accommodates cell-to-cell movement (Chay et al., 1996; Schmitz et al., 1997). Yet there are three luteoviruses, almond associated luteovirus 1 (AlLV1; Khalili et al., 2020), Red clover-associated luteovirus (RCaV; Lenz et al., 2018) and (Nectarine stem pitting associated virus (NSPaV; Bag et al., 2015) that do not encode readily identifiable MPs.

Here we describe a new species in the genus *Luteovirus* infecting blueberry that is phylogenetically related to and shares many of the attributes of the three aforementioned luteoviruses. Seven isolates of the virus, tentatively named Blueberry virus L (BlVL), were sequenced and primers were developed to assess the prevalence of the virus in five U.S. states; where the virus was found to infect almost 80% of all plants tested.

## 2 Materials and methods

### 2.1 Genome sequencing

Luteovirus-like sequences were first identified in 2017 after screening blueberry accessions for viruses using high throughput sequencing (HTS, Villamor et al., 2019). For further characterization, total RNA of several blueberries was submitted for HTS using Illumina HiSeq2500. VirFind (Ho and Tzanetakis, 2014) identified twelve luteovirus-like contigs in sample 2017-FRV-NGS41 collected in Oregon. Preliminary mapping of these contigs to the NSPaV genome identified regions of the genome not covered by the contigs. cDNA was synthesized with Maxima H Minus Reverse Transcriptase (Thermo Scientific) with random hexamer primers as per manufacturer's protocol. PCR primers which spanned the gaps between the contigs were designed (Supplementary Table 1) and these regions were amplified with Genescript Taq DNA polymerase and Sanger sequenced. The contigs and PCR amplicons resulted in a 4223 nucleotide (nt) long sequence missing the genome ends. All HTS reads were remapped to this sequence with an average of 14x coverage. The 5′ terminus was determined using the 5′ RACE System, (version 2, Thermo Fisher Scientific) whereas for the 3′ terminus the Poly(A)-tailing kit (Thermo Fisher Scientific) was used as per the manufacturer's instructions (primer sequences available in Supplementary Table 1). Amplicons of each terminus were cloned into pCR 2.1 TOPO vector (Thermo Fisher Scientific) and at least five clones for each region were sequenced.

An additional six near full genomes were amplified from Oregon samples (Supplementary Table 2). Reverse transcription was carried out with primer BlVL-R and Maxima H Minus Reverse Transcriptase (Thermo Scientific) as per manufacturers protocol with the optional 65 °C incubation step. Followed by PCR with Kapa HiFi polymerase and associated Fidelity buffer (Kapa Biosystems KK2102, protocol 95 °C_3min_(98 °C 20 s, 70 °C 15 s_,_ 72 °C _4._5 min)_40x_ 72 °C 8 min) using primers which bind within the 5′ and 3′ UTRs (Supplementary Table 1). Single plasmid clones were sequenced at Plasmidsaurus, using Oxford Nanopore Technology (www.plasmidsaurus.com) with coverage that exceeded 700x. All isolates were analyzed with Geneious Prime 2022.1.1 (https://www.geneious.com).

### 2.2 Genome analyses and phylogenetics

Analyses included isolates of 19 genomes, with REFSEQ isolates used where available; the 13 luteoviruses accepted by ICTV and six putative unclassified members of the genus: almond luteovirus 1 (AlLV1), peach associated luteovirus (PaLV) and four *Prunus*-infecting species (Khalili et al., 2023). NCBI ORFfinder and alignments to other luteoviruses was used to identify BlVL ORFs. Sequence Demarcation Tool v1.2 (SDT) (Muhire et al., 2014) with the MUSCLE alignment option, was used to calculate the pairwise nucleotide (nt) and amino acid (aa) identity of the genome and proteins as well as a dataset containing the eight conserved polymerase motifs present across the positive-strand RNA viruses (Koonin, 1991). Each dataset was aligned with MUSCLE, tested for the best-fit model, and maximum likelihood phylogenetic trees with 1000 bootstrap replicates were constructed within MegaX (Kumar et al., 2018). Branches with low bootstrap values (<60%) were collapsed in TreeGraph2 and unrooted trees were visualized in FigTree v1.4.4 (Stöver and Müller, 2010), (http://tree.bio.ed.ac.uk/software/ figtree/). The six near-full BlVL genomes were aligned with the reference sequence, with genome trees and percentage pairwise identities calculated as above.

ORF3a is present in the majority of luteoviruses, yet it is difficult to identify due to alternative start codons and its small size. Therefore, an alignment-based method was used in MegaX, with the sequence region between ORF2 and the start of ORF3, for all reference species, in all (+) frames with known P3a used as reference.

Other ORFs were further analyzed for evidence of transmembrane helices (TMH). All identifiable ORFs of apple luteovirus 1 (ALV1)*,* AlLV1, BlVL, NSPaV, Prunus mahaleb-associated luteovirus (PmaLV) and RCaV greater than 75 nt including those with alternative start codons (as identified in ORFfinder), were uploaded into TMHMM – 2.0 (Krogh et al., 2001). In addition, datasets of the putative TMH ORFs from 18 NSPaV and the seven BlVL isolates were compiled. For the NSPaV and BlVL datasets, the TMH sequence had gaps incorporated at the start and end to anchor the smaller TMH ORF relative to the corresponding expressed ORF. Sequences were analyzed for evolutionary selection using FUBAR within SelectionMap v1.0 (Stenzel et al., 2014). SelectionMap allows for each ORF to be analyzed for selection individually while providing a visual map of selection sites. RNA fold (Gruber et al., 2008; Lorenz et al., 2011) was used to identify the cap-independent translation elements in all BlVL genomes.

### 2.3 Virus prevalence

Samples from five U.S. states: Michigan (*n* = 194), New Jersey (*n* = 176), Pennsylvania (*n* = 87), Washington (*n* = 87) were collected in 2015–2016 as part of the Martin and Tzanetakis (2018) study, with samples from Oregon (*n* = 20) collected in 2019 as part of a different study (Table 2). Samples were collected randomly across fields and symptom information is largely unavailable. Total nucleic acids were extracted from leaf tissue as per Poudel et al. (2013) and stored at −80 °C. As samples were tested for BlVL and other blueberry viruses (data not shown), reverse transcription was carried out using both dT and random hexamer primers as described in Thekke Veetil et al. (2016) and screened with the NADH dehydrogenase ND-2 β subunit primers to evaluate the quality of cDNA (Tzanetakis et al., 2007). Screening primers BlVL3692F (5′-TGGATGGCGCGTGAGTATTC-3′)/BlVL4473R (5′-CCTTTCGGTAGCAACAATACC-3′) were developed and amplify a 782 nt region close to the 3′ end of the genome. The reaction consisted of 400 µM dNTPs, 400 nM of each of the screening primers, 10X *Taq* buffer, 0.5 U *Taq* DNA polymerase (Genscript, NJ) and molecular grade water to 25μl. PCR protocol of 94 °C_2min_ (94 °C_20 s_, 70 °C_20 s_,72 °C_40 s_)_40cycles_72 °C_8 min_. Bidirectional Sanger sequencing of a subset of these positive samples was carried out. For each sample, the reads were trimmed and assembled into consensus sequences with Geneious Prime (2022.1.1) recommended parameters (0.05 error probability trim, consensus threshold highest quality 60%). The BlVL3692F/BlVL4473R sequences were further trimmed within the primer sites. The seven full genome isolates were trimmed to the same region and the percentage pairwise identities for all 286 samples were calculated in SDT as above.

## 3 Results

### 3.1 BlVL genome architecture

The reference isolate of BlVL was sequenced from 2017-FRV-NGS41 and deposited under GenBank accession OQ686746. An additional six near full genomes were sequenced and deposited in GenBank under accessions OQ686747-OQ686752 (Supplementary Table 2).

**Fig. 1:**
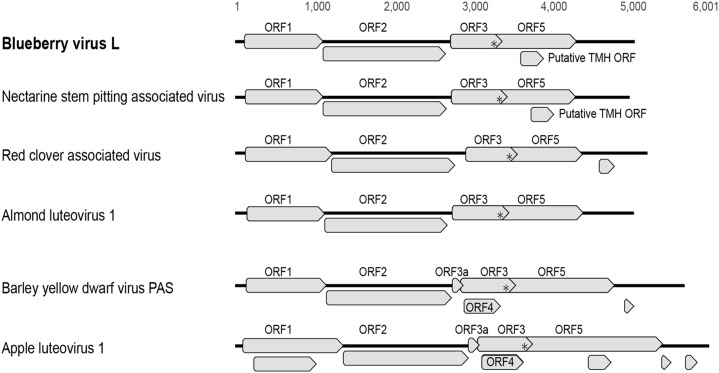
Cartoon representation of genomes of select luteovirus species. Genomes and ORFs are to scale. *represents stop codon in readthrough domain.

The full genome of BlVL is 5054 nt long and has a similar genome architecture to NSPaV with four canonical ORFs; ORF1 nt_119–1114_, ORF1-ORF2 nt_119–1111,1111–2673_, ORF3 nt_2732–3292_, ORF3-ORF5 nt_2732–4327_ (Fig. 1). BlVL and NSPaV also contain a putative ORF in the same sequence region as ORF5, which for BlVL is located between nt_3618–3914_. The expression of the P1-P2 protein occurs through a −1 frameshift in luteoviruses and is predicted in the heptanucleotide sequence GGNUUUU (Domier et al., 2002), which in BlVL is GGGUUUU_1105–1111_. The readthrough of the ORF3 stop codon (nt_3290–3292_) results in the fusion CP-RTD. Like AlLV1, NSPaV and RCaV, no putative MPs were identified in BlVL. The P3a-like protein was also not identified in BlVL, AlLV1, NSPaV, RCaV, or PmaLV. However, PmaLV does encode the P4 movement protein (Khalili et al., 2023).

Initiation of luteovirus translation occurs through the complementary binding of a conserved stem loop at the 5′ end of the gRNA and at the 5′ end of the subgenome, to the BYDV-like cap-independent translation element (BTE) located in the 3′ UTR (Guo et al., 2001; Wang et al., 1997). The subgenome contains the ORFs coding for P3/P5 and the two movement proteins (when present). In BlVL, the BCL sequence UGACA is present at nt_100_ and nt_2630_ with the nucleotides either side forming the stem. The complementary stem-loop III (SL-III) sequences of the BTE, which binds to BCL, is UGUCA in all six 2020 isolates and CGUCA in 2017-FRV-NGS41 at nt_4497_. Both UGUCA and -GUCA are BTE SL-III loop sequences present in a number of barley yellow dwarf virus isolates (Miller et al., 2015; Shen and Miller, 2004).

The helicase motifs I/Ia, II—IV, and VI, have been previously identified in the luteoviruses barley yellow dwarf virus-PAV BYDVPAV and soybean dwarf virus (Habili and Symons, 1989). Motifs I/Ia-III are located in the P1 region of the replicase and IV and VI embedded within P2. The majority of the luteovirus species analyzed here, including BlVL do not include identifiable P1 motifs as there is little aa sequence consensus in the alignments in this region. All luteovirus species contain the P2 helicase motifs IV and VI; motif IV is highly conserved across all luteovirus species with BlVL present as RRVFTVGKG_396–404_ whereas motif VI is present in BlVL as RYNVEVGRRLKFNEKK_500–515_.

### 3.2 Percentage pairwise identities

**Fig. 2:**
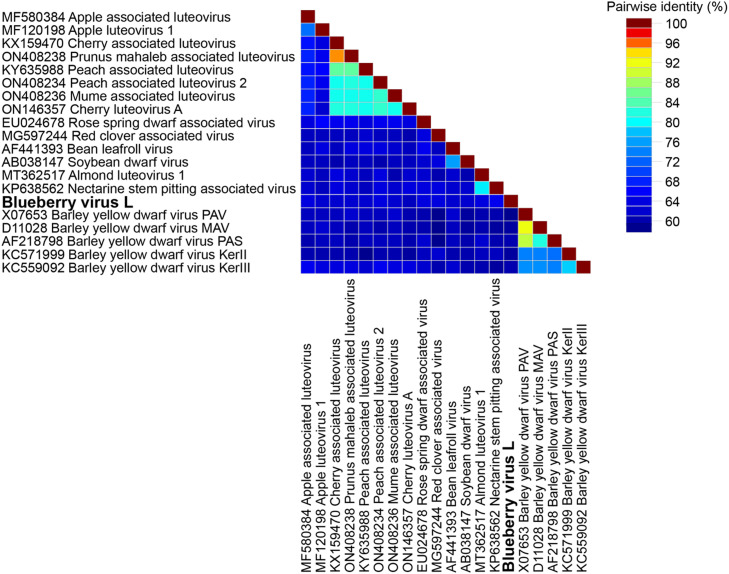
Percentage pairwise identity matrix of the full genomes of luteovirus species. Each colored square shows the percentage pairwise nucleotide identities between two isolates.

The BlVL reference isolate OQ686746, shares 58.6–62.6% nt full genome pairwise identity with other luteoviruses, with NSPaV (KP638562) being the most similar and barley yellow dwarf virus PAV (XO7653) the least (Fig. 2: Table 1: Supplementary Data 1). All BlVL isolates share 93.1–99.2% pairwise nucleotide identity, with isolates 2020–3-OR and 2020–5-OR being the most divergent (Table 1; Supplementary Data 1). When comparing the different BlVL proteins to luteovirus orthologs, the replicase (P1-P2) has the highest aa identity of all the proteins, sharing (52.9–61.1%) to all other luteoviruses, with highest identity of 61.1% seen between BlVL and both the apple associated luteovirus (AaLV) and RCaV. The other proteins P1, P3, P3-P5 are all <50% aa identity (Table 1).

**Table 1:** Percentage pairwise identity overview for luteovirus species. Full matrices of all percentage pairwise identities are in Supplementary Data 1. Putative TMH – open reading frame within BlVL with evidence of encoding a transmembrane helicase. AaLV – apple associated luteovirus, AlLV1 – Almond associated luteovirus 1, RCaV – red clover-associated luteovirus, NSPaV – Nectarine stem-pitting associated virus, PaLV - Peach associated luteovirus.

Pairwise comparison between isolates	Full Genome	Helicase-like (P1)	Replicase (P1-P2)	Polymerase conserved region	Capsid protein (P3)	Capsid protein – RTD (P3-P5)	Putative TMH
	% PI nt	% PI aa	% PI aa	% PI aa	% PI aa	% PI aa	% PI aa
Luteovirus reference isolates	58.4%−88.6%	30%−97.6%	49.7%−97.5%	65.3%−98.3%	29.7%−87.4%	31.4%−87.8%	
BlVL reference vs all luteoviruses (highest identity to BlVL)	58.6%−62.6% NSPaV	34.5%−46.5% AaLV	52.9%−61.1% RCaV & AaLV	70.7%−79.5% RCaV	34.9%−47.3% AlLV1 & PaLV	34.7%−50.0% NSPaV	
All seven BlVL isolates	93.1%−99.2%	88.5%−100%	93.5%−99.8%		94.6%−100%	96.8%−100%	74.5%−96.9%

Across all luteoviruses, the replicase share 49.7–97.5% aa identity, with the highest identities seen amongst BYDV species. Due to low similarities of the replicase across the majority of the species, a smaller conserved region of the replicase was analyzed This region spans motif I-VIII, motifs which were previously identified as conserved across the positive strand viruses (Koonin, 1991). This region is located in the P2 portion of the luteovirus replicase, with the BlVL isolate motifs as follows I: FLKMEKHL/HM-CK _477–487_, II: PRLICPRTKRYNVEVGRRLKFN_491–512_, III: VLSGYDSFSVGRIIAKKWN_570–588_, IV: KTPVAIGVDASRFD_550–563_, V: EGHRMSGDINTSMGNKLVMCGMMHNYFRE_613–641_, VI: AELCNNGDDCVI_636–657_, VII: LEFCQSRPVC_693–702_ and VIII: MVRRPDS_709–715_, with locations relative to the start of the replicase. This region containing motif 1–8 shares 65.3–98.3% amongst all luteoviruses, with BlVL isolates sharing highest identity (79.5%) to RCaV.

### 3.3 Phylogenetics

**Fig. 3:**
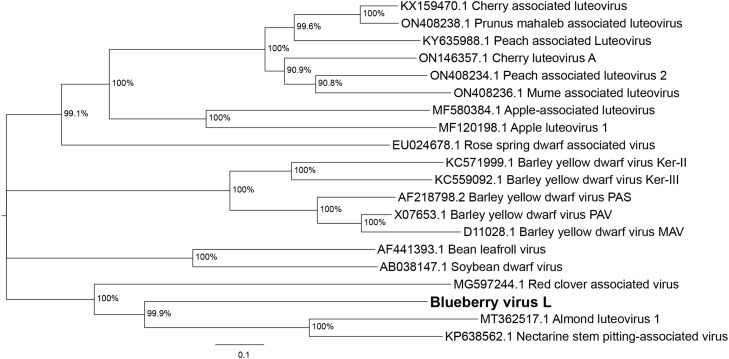
Unrooted Maximum likelihood (GTR+GI model) nucleotide phylogenetic tree of full genomes of luteovirus species. Branches show Bootstrap support (1000 replicates), with branches with <60% collapsed.

A maximum likelihood (ML) full genome nucleotide tree (model – General Time Reversable model with rates among sites Gamma distributed with Invarient sites (GTR+GI)) shows the clustering of the BlVL relative to other luteoviruses (Fig. 3). Full genomes of the luteoviruses fall into four clades, with the BlVL clustering with RCaV, AlLV1 and NSPaV. As the luteovirus classification within the *Tombusviridae* is based on the replicase, a ML phylogenetic tree (model – Le Gascuel model with rates among sites Gamma distributed (LG+G)) of the fusion protein was also constructed (Supplementary Fig. 1), which shows similar clustering.

### 3.4 Putative proteins with transmembrane helicase signals

Three of the four viruses without identifiable movement proteins, AlLV1, BlVL and NSPaV showed evidence of putative proteins containing transmembrane helicase (TMH) signals (six, three, three ORFs respectively). All three viruses contain a putative TMH ORF in a similar location, embedded within ORF 5; BlVL (99aa), NSPaV (86aa) and AlLV1 with two ORFs (80aa and 55aa). The previously characterized ALV1 contains additional ORFs, one of which is also in the ORF5 nucleotide region (Liu et al., 2018) (Fig. 1), however this ORF did not show evidence of TMH. PmaLV which encodes P4 but not P3a, also contains a putative ORF with a TMH signal embedded at the start of ORF 5. As both AlLV1 and PmaLV have only one isolate available no further analysis was undertaken.

The putative TMH ORFs within ORF5 for additional BlVL and NSPaV isolates were further analyzed. Two BlVL isolates, 2020–1-OR and 2020–5-OR had slightly smaller TMH ORFs (83aa and 96aa). All NSPaV and BlVL TMH ORFs utilize an alternative start codon, with TTG for all NSPaV and 2020–1-OR, and CTG for the rest of the BlVL. TMHMM-2.0 predicted one (BlVL *n* = 4, NSPaV *n* = 2) or two (BlVL *n* = 2, NSPaV *n* = 11) transmembrane helices for the majority of the isolates, however for a small number (BlVL *n* = 1, NSPaV *n* = 5) fell below the probability threshold (Supplementary Data 2). The putative orthologous ORFs show low aa identity between the two species (27–63%) and within species (75–99% for BlVL and 79–100% for NSPaV).

### 3.5 Selection analyses of putative TMH ORFs and P5 region

**Fig. 4:**
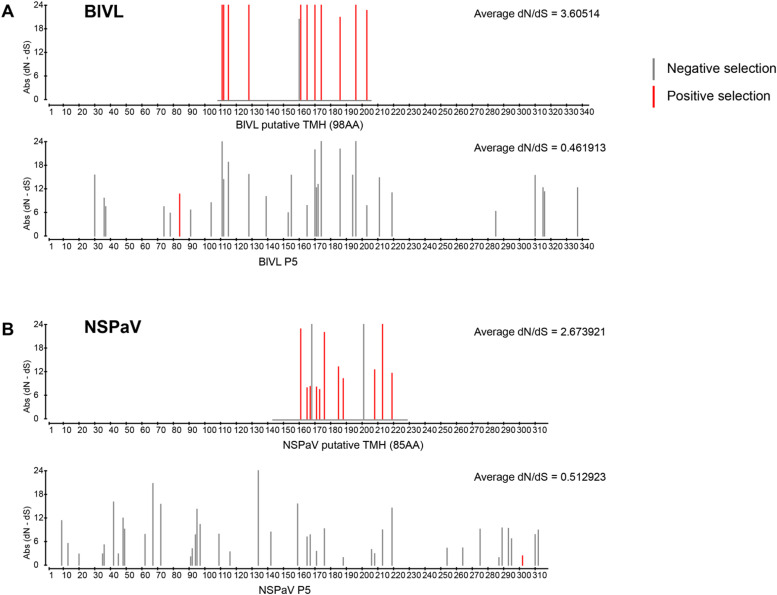
Site specific selection detected by FUBAR in the blueberry virus L (BlVL) (A) and nectarine stem-pitting associated virus (NSPaV) isolates (B). Selection was identified for each protein, the putative proteins with evidence of transmembrane helices (TMH), and the CP-RTD (P5) region. Codon sites evolving under positive selection (red) and negative selection (gray) are shown with absolute non synonymous/synonymous substitutions (dN/dS), and average dN/dS for each protein.

Both BlVL and NSPaV putative TMH proteins show multiple sites are evolving under positive selection with the average dN/dS (non-synonymous/synonymous substitutions) showing overall positive selection for BlVL the average dN/dS=3.61 and for NSPaV the average dN/dS=2.67 (Fig. 4). The P5 region however shows overall negative selection for both BlVL and NSPaV, with average dN/dS=0.46 and dN/dS=0.51 respectively. Comparison on the approximate locations of the TMH region over the corresponding P5 region, shows differences in the two species. For BlVL (Fig. 4), there are high numbers of sites evolving under negative selection in the P5 region located in the corresponding TMH region. Whereas with NSPaV the sites evolving under negative selection are more uniformly distributed across P5 (Fig. 4).

Additional UGACA sequences, potential BCL sequences, are present within and after the TMH ORF, at nt_3817_ (seven isolates) and nt_4177_ (six isolates), respectively. However, neither of these are predicted to fold into stem-loop structures. ORF4 (when present) is translated via leaky scanning (Dinesh-Kumar and Miller, 1993) yet the distance of the TMH ORF from ORF3 start codon is likely too distant (887 nt).

BlVL Prevalance

BlVL was found in >50% of samples in each of the localities. Samples collected in Pennsylvania, New Jersey and Washington had an incidence of 54%, 75% and 84% respectively. Both Oregon and Michigan show especially high incidence with 90% in Oregon and 93% in Michigan (Table 2). Sanger sequencing of the positive samples resulted in 279 sequences ranging from 612 to 743 nt in length (GenBank OQ709486-OQ709764). These isolates along with the same region from the seven BlVL isolates showed significant sequence diversity, with percentage pairwise identities of 84.8–100%, with the lowest identity seen between sequences from New Jersey (NJ) and Michigan (MI) (NJ1_H11 to MI2_E4) and (MI2_E4 to NJ2_H3) (full matrix in Supplementary Data 1). Cultivars that tested positive for BlVL include: Berkley, Bluecrop, Blueray, Bluetta, Collins, Darrow, Draper, Duke, Earliblue, Elliott, Herbert, Jersey, Liberty, Nelson, Old Jersey, and Toro.

## 4 Discussion

Here we describe blueberry virus L, a novel luteovirus. The BlVL genome architecture is similar to AlLV1, NSPaV and RCaV; all having four canonical ORFs and without identifiable MPs. The ICTV species demarcation for luteoviruses is that of a virus where any protein shows >90% aa identity (King et al., 2011); with all BlVL ORFs being well below this threshold when compared to the other luteovirus species.

The seven sequenced BlVL isolates show 93.1–99.2% nt identity yet three share ∼89% aa identity in P1, when compared to the others. While this is lower than the ICTV species demarcation, the rest of the proteins share >93% aa identity. A recent update of the *Betaflexiviridae* taxonomic criteria has been proposed; where in instances that the aa identity of one protein is close to the cutoff threshold, (which in BlVL case the lowest aa identity is 88.5% with a cutoff value of 90%) then the aa identity of an additional protein is considered (Silva et al., 2022). Therefore, taking into consideration all other aa% identities of BlVL, all isolates presented here are the same species.

When comparing the BlVL genome to other luteoviruses, BlVL has the highest percentage pairwise identity to AlLV1, NSPaV and RCaV, yet the encoded proteins do not always show highest identities to these three viruses (Table 1). P1 has highest pairwise identity to AaLV, and the replicase (P1-P2) and capsid protein share equal percentage pairwise identity with RCaV and AaLV, and AlLV1 and peach associated luteovirus (PaLV) respectively. Phylogenetically, neither AaLV or PaLV full genomes cluster near the BlVL clade suggesting recombination may have occurred across these (or ancestral viruses) during the evolution of BlVL, with evidence of recombination events identified in other luteoviruses (Pagán and Holmes, 2010). Another hypothesis would be asymmetrical evolution based on the hosts the viruses infect at the time.

Based on the high prevalence of BlVL and a survey of *Prunus* species that identified NSPaV in higher incidence than other prunus-infecting luteoviruses (Khalili et al., 2023), suggests that the lack of identifiable movement-associated proteins (P3a, P4) does not have a major impact on virus prevalence. We have identified a putative ORF which contains evidence of transmembrane helices, which are indicative of a protein involved in movement (Krogh et al., 2001; Morozov and Solovyev, 2020). Yet there is no identifiable mechanism on how this ORF is translated based on current knowledge of the luteovirus expression strategies. Further complexity is added when considering the selection analyses. Seemingly unfavorable, asymmetric evolution of expressed viral genes is not unusual, with a study finding half of the viruses tested were evolving asymmetrically (Pavesi, 2019). So, while it is unclear how or if the identified putative ORFs are expressed, the fact that isolates of both NSPaV and BlVL contain a putative ORF with evidence of TMH in a similar genome region and both show high positive selection while being constrained by negative selection of the overlapping ORF5, warrants further investigation.

BlVL was found in high prevalence in survey samples collected across five US states suggesting that either this virus has been circulating undetected for a long period of time, is moving through propagation material, and/or it spreads rapidly in the field. The movement of propagation material is always of concern yet the high diversity of the 286 samples sequenced suggests multiple introductions, rather than the movement of clonally propagated material. A previous study of blueberry viruses showed regional differences in the most prevalent species, with the incidence of the most prevalent virus varying from 5%−45% in each region and the total incidence across the US of a single species at 21% (Martin and Tzanetakis, 2018). Comparatively, BlVL has higher incidence per state and total incidence in the US making it the most widespread blueberry virus in the US to date.

The high diversity and wide distribution of BlVL in commercial plantings suggests a long association of this virus, with US blueberry cultivars first released in the 1920s with many early cultivars still used today (Mainland, 2012). It also suggests that this virus does not cause significant symptoms in blueberry in single infections since it has not been identified prior to HTS analysis of blueberry. However, there is a high likelihood that these samples contain mixed infections with BlVL as these samples have previously been tested for a number of other viruses (Martin and Tzanetakis, 2018). It is unknown what impact mixed infections have on blueberry but as a long-cropped plant, the presence and accumulation of multiple viruses is a concern. Further studies into movement, symptomology and mixed infections of BlVL are needed to better understand this newly characterized virus.

## Funding

This work was partially funded by the 10.13039/100000199USDA under agreements AP20PPQS&TOOC170, AP21PPQS&T00C011 and USDA-NIFA Hatch project 1002361.

## Author statement

The authors declare that they do not have any conflicts of interest.

##  

**Table 2:** Prevalence of Blueberry virus L (BlVL) in survey samples collected as part of the Martin and Tzanetakis (2018) study. Survey samples were screened using primers BlVL3691F/ BlVL4475R. States with multiple survey plates also show overall Incidence. NJ – New Jersey, MI – Michigan, WA – Washington, PS – Pennsylvania, OR – Oregon.

State	Collection Year	PCR positive	Total samples	
NJ	2015	178	237	75%
MI	2015	180	194	97%
WA	2017	73	87	84%
PA	2015	47	87	54%
OR	2019	18	20	90%
		**496**	**625**	**79%**

Supplementary Table 1: Oligonucleotide primers used in the study.

Supplementary Table 2: Blueberry virus L genome sequencing isolates. *Illumina sequencing coverage. ^#^Single plasmid clones, commercially sequenced with Oxford Nanopore Technology.

Supplementary Data 1: Percentage pairwise identity matrices calculated by SDT for all datasets of the luteovirus species and BlVL.

Supplementary Data 2: Graphical output of evidence of the presence of transmembrane helices for each isolate detected with the TMHMM-2.0 program.

Supplementary Figure 1: Unrooted Maximum Likelihood (LGG model) amino acid phylogenetic tree of replicase (P1-P2) of luteovirus species. Bootstrap (1000 replicates) support branches with <60% are collapsed.

## Declaration of Competing Interest

The authors declare that they do not have any conflicts of interests associated with the research presented here

## Data Availability

Data will be made available on request.

## Appendix Supplementary materials

Image, application 1

Image, application 2

Image, application 3

Image, application 4

Image, application 5
