# Supplementary material for: Genomic analyses of a widespread blueberry virus in the United States
Source: Virus Res. 2023 Jun 7;333:199143. doi: 10.1016/j.virusres.2023.199143 (PMC10352716; doi:10.1016/j.virusres.2023.199143)
Supplement: Supplementary file 1 [file mmc1.docx]

Supplementary Table 1: Oligonucleotide primers used in the study

|  | **Name** | **Sequence** **(5'-3')** |
| --- | --- | --- |
| Amplification of sequences | BlVL-813F | TGGGTTCGCCGTATTCGTGACG |
| between HTS contigs | BlVL-3763R | TGCGACAACCCGAATACTCACGC |
|  | BlVL-1429F | AACCTATAGTCCGATGGAGCTTGC |
|  | BlVL-1883F | CTCGATCAACTGCTGGACTGGC |
|  | BlVL-3574F | GCTCTCCGGGCAAGTACTCAG |
|  | BlVL-4176R | GGCCTGTCTACTACCGAAGCAG |
|  | BlVL-4155F | CTGCTTCGGTAGTAGACAGGCC |
|  | BlVL-4975R | GCTTGAGAAACGCACGAACAGG |
|  | BlVL-4670F | GTCCAGCTTCCACGAGTTGGC |
| 5' RACE | BlVL-907R | TCATCCGCAGAGAGACGGGC |
|  | AAP | GGCCACGCGTCGACTAGTACGGGIIGGGIIGGGIIG |
| 3' RACE | dT primer | GGCCACGCGTCGACTAGTACTTTTTTTTTTTTTTTTTT |
|  | Adapter | GGCCACGCGTCGACTAGTAC |
|  | BlVL-4670F | GTCCAGCTTCCACGAGTTGGC |
| Full genome RT | BlVL-R | GGGCGCGTACGAACGCGACG |
| Near full genome primers | BlVL-5utrF | GGCTAGAGGTACAGCATACATCTCTGG |
|  | BlVL-3utrR | CCCCCTTTTTAACGTGGAAGAGCC |
| NADH dehydrogenase ND-2 subunit β subunit | NADH-F | GGACTCCTGACGTATACGAAGGATC |
|  | NADH-R | AGTAGATGCTATCACACATACAAT |
| BlVL screening primers | BlVL-3692 F | TGGATGGCGCGTGAGTATTC |
|  | BlVL-4473 R | CCTTTCGGTAGCAACAATACC |
