# Supplementary material for: Genomic analyses of a widespread blueberry virus in the United States
Source: Virus Res. 2023 Jun 7;333:199143. doi: 10.1016/j.virusres.2023.199143 (PMC10352716; doi:10.1016/j.virusres.2023.199143)
Supplement: Supplementary file 2 [file mmc2.docx]

Supplementary Table 2: Blueberry virus L genome sequencing isolates. *Illumina sequencing coverage. ^#^Single plasmid clones, commercially sequenced with Oxford Nanopore Technology.

| Sample | State | Cultivar | Sequencing coverage | Collection year | Accession number |  |  |  |  |  |
| --- | --- | --- | --- | --- | --- | --- | --- | --- | --- | --- |
| 2017-FRV-NGS41 | OR | unknown | ***Average 14x | 2017 | OQ686746 |  |  |  |  |  |
| 2020-1-OR | OR | Liberty | ^#^1180x | 2019 | OQ686747 |  |  |  |  |  |
| 2020-2-OR | OR | Draper | ^#^4800x | 2019 | OQ686748 |  |  |  |  |  |
| 2020-3-OR | OR | unknown | ^#^3830x | 2019 | OQ686749 |  |  |  |  |  |
| 2020-5-OR | OR | Draper | ^#^4100x | 2019 | OQ686750 |  |  |  |  |  |
| 2020-6-OR | OR | Draper | ^#^7380x | 2019 | OQ686751 |  |  |  |  |  |
| 2020-7-OR | OR | Draper | ^#^701x | 2019 | OQ686752 |  |  |  |  |  |
