# Supplementary figures and images for: Genomic analyses of a widespread blueberry virus in the United States
Source: Virus Res. 2023 Jun 7;333:199143. doi: 10.1016/j.virusres.2023.199143 (PMC10352716; doi:10.1016/j.virusres.2023.199143)

## Slide 1
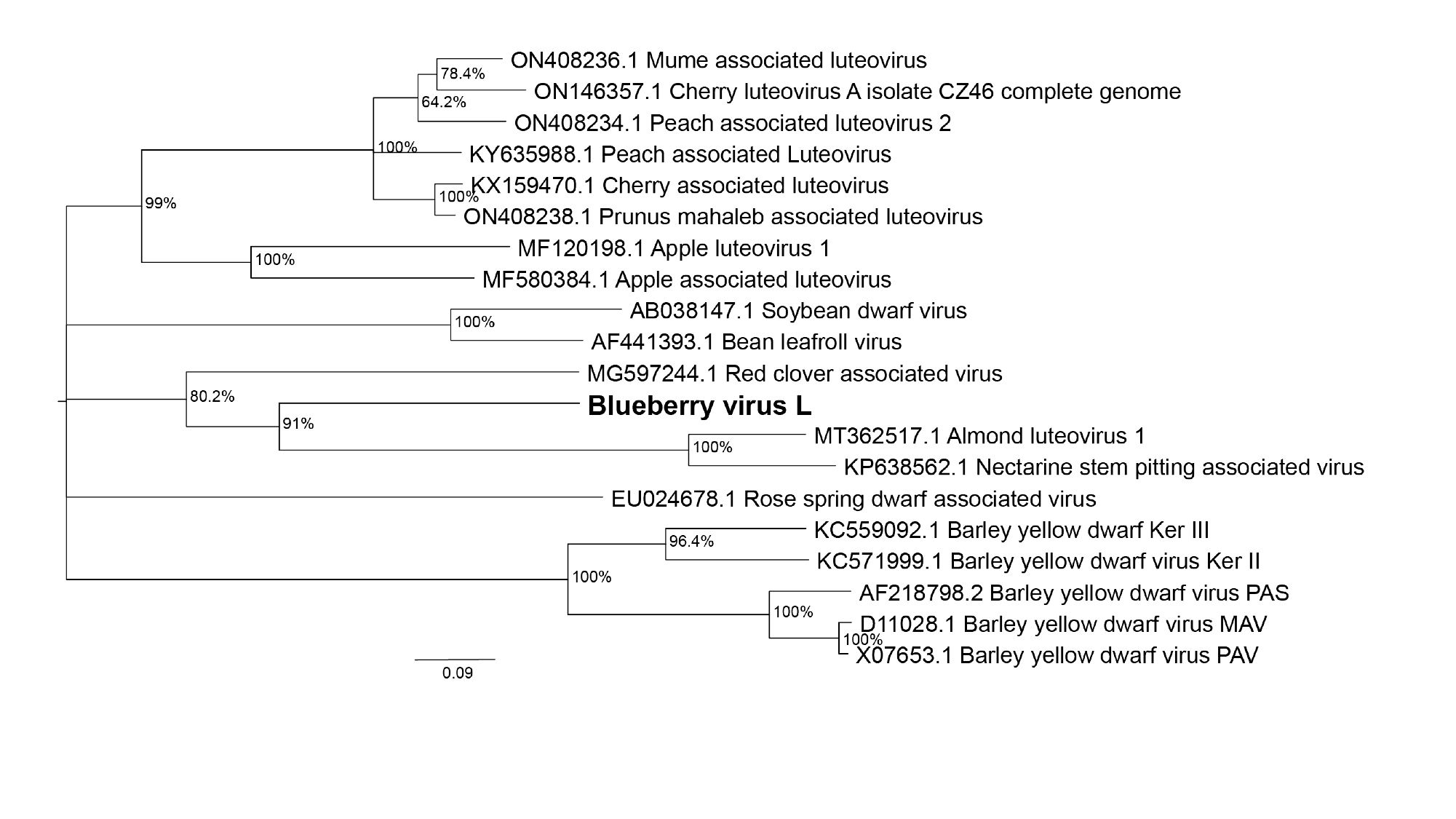

Supplement: Supplementary file 5 [file mmc5.pptx]
